# Role of ferroptosis in the pathogenesis of heart disease

**DOI:** 10.3389/fphys.2024.1450656

**Published:** 2024-09-10

**Authors:** Sulail Fatima, Haiyan Zhou, Yi Chen, Qinghang Liu

**Affiliations:** Department of Physiology and Biophysics, University of Washington, Seattle, WA, United States

**Keywords:** ferroptosis, iron overload, lipid peroxidation, signaling, heart disease

## Abstract

Ferroptosis is a new form of regulated necrosis characterized by iron-dependent lipid peroxidation, leading to irreparable lipid damage, membrane permeabilization, and necrotic cell death. Ferroptosis has recently been implicated in the pathogenesis of multiple forms of heart disease such as myocardial infarction, cardiac hypertrophy, heart failure, and various cardiomyopathies. Important progress has also been made regarding how ferroptosis is regulated *in vitro* and *in vivo* as well as its role in cardiac homeostasis and disease pathogenesis. In this review, we discuss molecular mechanisms that regulates ferroptosis in the heart, including pathways leading to iron overload and lipid peroxidation as well as the roles of key organelles in this process. We also discuss recent findings pertaining to the new pathogenic role of ferroptosis in various forms of heart disease as well as genetic and pharmacologic strategies targeting ferroptosis in the heart.

## 1 Introduction

Apoptotic and/or necrotic cell death has been implicated in multiple forms of heart disease, including ischemic myocardial injury, pathological remodeling, myocarditis, various forms of cardiomyopathy, and drug-induced cardiotoxicity (Del Re et al., 2019). Apoptosis is the most renowned form of regulated cell death mediated by death receptor or mitochondria dependent signaling pathways, which is characterized by cytosolic shrinkage, membrane blebbing, chromatin condensation, and DNA fragmentation, without loss of plasma membrane integrity (Danial and Korsmeyer, 2004). In contrast, necrosis had long been regarded as an unregulated process triggered by excessive pathological stress, characterized by cell swelling, plasma membrane rupture, cell lysis, and inflammatory response (Edinger and Thompson, 2004). However, this notion has been overturned by emerging evidence revealing that necrosis can also occur in a highly regulated and genetically controlled manner, termed “regulated necrosis”. Indeed, a number of regulated necrotic cell death modalities have recently been identified, including ferroptosis, necroptosis, pyroptosis, parthanatos, mitochondria-mediated necrosis, and other regulated necrotic processes (Del Re et al., 2019).

Ferroptosis is a newly identified form of regulated necrosis characterized by iron-dependent lipid peroxidation, leading to irreparable lipid damage, membrane permeabilization, and necrotic cell death (Dixon et al., 2012; Stockwell et al., 2017). Iron overload is a hallmark of ferroptosis, which promotes lipid peroxidation by producing hydroxyl and alkoxyl radicals through the Fenton reaction (Papanikolaou and Pantopoulos, 2005). Moreover, iron can also participate in enzymatic lipid peroxidation by promoting the activation of arachidonate lipoxygenase (ALOX) (Pu et al., 2022). Ferroptosis, regardless of the mechanisms of induction, is effectively inhibited by iron chelators, such as deferoxamine, indicating that iron is critically involved in the execution of ferroptosis. Moreover, cellular susceptibility to ferroptosis is closely regulated by iron metabolism, including its import, export, utilization, and storage (Tang D. et al., 2021). Accumulation of lipid peroxidation products is another hallmark of ferroptosis. Glutathione peroxidase 4 (GPX4), a glutathione (GSH)-dependent selenoenzyme, plays a crucial role in preventing ferroptosis by converting toxic lipid hydroperoxides to nontoxic lipid alcohols (Friedmann Angeli et al., 2014; Yang et al., 2014). Failure of GPX4 to clear lipid reactive oxygen species (ROS) leads to overwhelming lipid peroxidation and ferroptotic cell death (Friedmann Angeli et al., 2014; Yang et al., 2014). Apoptosis-inducing factor mitochondria-associated 2 (AIFM2, also known as FSP1) has been identified as another key antioxidant protein that acts parallel to GPX4 in suppressing phospholipid peroxidation and ferroptosis (Bersuker et al., 2019; Doll et al., 2019). Moreover, the enzymes involved in the peroxidation of polyunsaturated fatty acids (PUFAs), such as acyl-CoA synthetase long-chain family member 4 (ACSL4), lysophosphatidylcholine acyltransferase 3 (LPCAT3), and ALOXs, also play important roles in the induction of ferroptosis (Dixon et al., 2015; Doll et al., 2017; Kagan et al., 2017).

**FIGURE 1:**
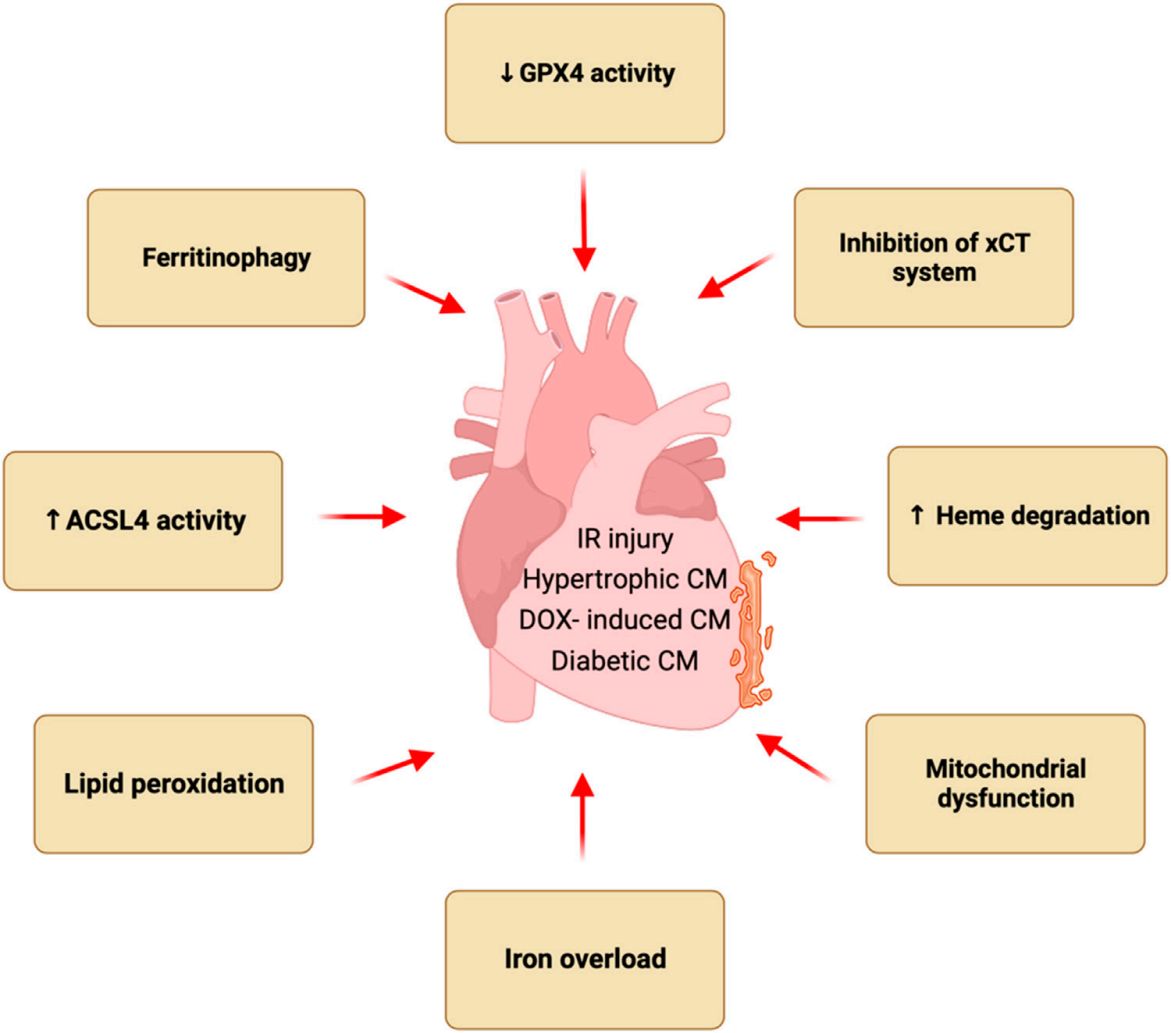
Ferroptosis in heart diseases. Ferroptosis has been implicated in the pathogenesis of multiple forms of heart disease such as myocardial infarction, cardiac hypertrophy, and various cardiomyopathies. Pathological stress induces ferroptotic cell death in the heart via multiple mechanisms such as iron overload, and lipid peroxidation, inhibition of xCT system, reduced GPX4 activity, increased ACSL4 activity, ferritinophagy, heme degradation, and mitochondrial dysfunction.

Ferroptosis has recently been implicated in the pathogenesis of multiple forms of heart disease such as myocardial infarction, cardiac hypertrophy, heart failure, and various cardiomyopathies. New mechanistic insights have also been obtained regarding how ferroptosis is regulated *in vitro* and *in vivo* as well as its role in cardiac homeostasis and disease pathogenesis (Figure 1). Here, we review recent findings pertaining to the new pathogenic role of ferroptosis in various forms of heart disease as well as genetic and pharmacologic strategies that target ferroptosis in the heart. Molecular and cellular mechanisms of ferroptosis, especially pathways leading to iron overload and lipid peroxidation as well as new roles of key organelles, have recently been elucidated. Emerging evidence also reveals that ferroptosis contributes to the pathogenesis of acute cardiac injuries as well as chronic diseases by inducing cell death, inflammation, and tissue remodeling.

## 2 Mechanism of ferroptosis

### 2.1 Iron overload

#### 2.1.1 Iron regulation

The absorption of diet iron involves several steps, including the uptake of iron from the intestinal lumen across the apical border of the villus and its transfer across the basolateral border to the plasma (Dev and Babitt, 2017). Extracellular iron in blood reversibly binds to transferrin (TF), a glycoprotein that is essential for the transport and cellular uptake of iron. Each transferrin molecule contains two binding sites for ferric ion. Iron-loaded transferrin is transported to the tissues, mainly erythroid marrow where it binds with transferrin receptor protein 1 (TFR1) and is internalized through clathrin-dependent endocytosis. The low pH environment in the endosome causes the release of ferric iron from the TF-TFR1 complex and, a transmembrane ferrireductase STEAP3 (six-transmembrane epithelial antigen of prostate) reduces ferric iron to ferrous iron. Next, DMT1 (divalent metal transporter 1) transports ferrous iron from the endosome into the cytosol (Bersuker et al., 2019). The carrier protein transferrin and TFR1 receptor are recycled back to the ECF and cell surface, respectively. Iron can be stored in the cytosol by the iron storage protein ferritin, which can chelate about 4,500 iron atoms (Chen et al., 2020). Iron enters the mitochondria via mitoferrin 1 and 2 where it participates in heme biosynthesis and hemoglobin production in developing erythroblasts (Shaw et al., 2006). Ferritin is also present in the mitochondria, termed mitochondrial ferritin (FTMT) (Santambrogio et al., 2007; Levi et al., 2021). Iron can be exported out of the cell by ferroportin-1 (FPN1, also known as SLC40A1) (Donovan et al., 2000; Azucenas et al., 2023). FPN1 is highly expressed in duodenal enterocytes, hepatocytes and macrophages (D’Anna et al., 2009).

In mammals, the iron regulatory proteins (IRPs; IRP1 and IRP2) are the central regulators of iron uptake, storage and export (Wang L. et al., 2019). In iron deficient states, IRPs bind to the iron response element (IRE) in the 3′UTR of target transcripts like TFR and DMT to stabilize the mRNA and increase translation of mRNA. At the same time, IRPs bind to the IRE in the 5′UTR of target transcripts such as ferritin and ferroportin to suppress translation of these proteins to combat iron deficiency. When iron levels are sufficient, the IRP system is under suppression. IRP1 contains Fe-S cluster which does not allow it to bind to IRE, and IRP2 is degraded by ubiquitin ligase which is sensitive to iron levels (Chen et al., 2020). Additionally, ferroportin levels are also regulated by hepcidin (Berezovsky et al., 2022). Hepcidin prevents iron efflux from the cells by binding to ferroportin and inducing endocytosis followed by the degradation (Charlebois et al., 2022).

#### 2.1.2 Mechanisms of iron overload

##### 2.1.2.1 Ferritinophagy

Excess iron within the cell is stored in ferritin to prevent iron-mediated lipid peroxidation and ferroptosis. Under conditions of iron deficiency or high iron demand, ferritin undergoes autophagic lysosomal degradation to increase the labile iron content within the cells. However, elevated autophagy of ferritin, termed ferritinophagy, can induce iron overload and ferroptosis. The nuclear receptor coactivator 4 (NCOA4) serves as a specific cargo receptor for transporting ferritin to lysosomes for autophagic degradation (Mancias et al., 2014). Autophagy-related 5 and 7 (Atg5 and Atg7, respectively) genes are also critical for the formation of autophagosome during the process of ferritinophagy (Hou et al., 2016; Wen et al., 2019). The intracellular NCOA4 levels are regulated by cellular iron load. In conditions of high cellular iron levels, HERC2 (ECT and RLD domain-containing E3 ubiquitin protein ligase 2) facilitates the ubiquitination of NCOA4, marking it for degradation via the proteasome. This degradation limits NCOA4 availability, thereby reducing its ability to transport ferritin to lysosomes. In contrast, during iron deprivation, HERC2’s hold on NCOA4 weakens, allowing a pool of NCOA4 to remain unubiquitinated. This liberated NCOA4 can then bind to ferritin, facilitating its transport to lysosomes for degradation, consequently releasing iron for cellular utilization (Liu et al., 2020). It has been shown that NCOA4-mediated ferritinophagy was activated following pressure overload, leading to ferrous iron overload, increased lipid peroxidation, cardiomyocyte death, and ultimately heart failure in mice (Ito et al., 2021). Suppression of ferritinophagy by NCOA4 silencing protected the cells from iron overload and ferroptosis (Fang et al., 2021; Santana-Codina et al., 2021).

##### 2.1.2.2 Heme degradation by heme oxygenase-1

Heme is a crucial component of various biological processes like oxygen transport, electron transport, metabolism of drugs and toxins and signal transduction (Seiwert et al., 2020). Heme oxygenase (HO-1), a 32-kDa protein encoded by the *Hmox1* gene, mediates the catabolism of heme into biliverdin, carbon monoxide (CO), and iron (Fe^2+^) (Cruse and Maines, 1988). Although HO-1 can elicit cytoprotective effects (Costa et al., 2020; Seiwert et al., 2020), excessive HO-1 activation can lead to iron overload, causing tissue damage and organ dysfunction (Miyamoto et al., 2022). In sickle cell disease, excess systemic heme has been shown to upregulate HO-1 expression and exacerbate iron overload, leading to cardiac ferroptosis and cardiomyopathy in mice (Menon et al., 2022). HO-1 upregulation has also been shown to promote iron overload in beta-thalassemia and anthracycline cardiotoxicity (Garcia-Santos et al., 2018; Fang et al., 2019). Importantly, a recent study showed that HO-1 silencing prevented simulated I/R-induced ferroptosis in cardiomyocytes (Miyamoto et al., 2022). Intriguingly, both pro- and anti-ferroptotic roles of HO-1 have been reported depending on cell types and pathological conditions (Chiang et al., 2018). To explain this discrepancy, accumulating evidence suggests that moderate activation of HO-1 elicits a cytoprotective effect whereas excessive and/or prolonged activation of HO-1 induces iron overload, leading to ferroptotic cell death (Chiang et al., 2018).

### 2.2 Lipid peroxidation

#### 2.2.1 Mediators of lipid peroxidation

Free PUFAs, crucial substrates in lipid peroxidation, are incorporated into phospholipids by two pivotal enzymes: ACSL4 and LPCAT3. Inhibition of ACSL4 and LPCAT3 diminishes the availability of substrates necessary for lipid peroxidation, thus enhancing resistance to ferroptosis (Li and Li, 2020; Xu et al., 2020; Cui et al., 2021). During ferroptosis, PUFA derivatives within cellular membranes, such as the endoplasmic reticulum, mitochondria, lysosomes, and plasma membrane, undergo lipid peroxidation either via non-enzymatic Fenton reactions or enzymatic processes (Chen et al., 2021; Von Krusenstiern et al., 2023). Several enzyme systems are involved in lipid peroxidation, such as xanthine oxidase, cytochrome P450, NADPH oxidase, cyclooxygenases (COX), and lipoxygenase (LOX), many of which are iron dependent. LOX are iron containing nonheme dioxygenases, encoded by six ALOX genes ALOX5, ALOX12, ALOX12B, ALOX15, ALOX15B, and ALOXE3, which play an important role in ferroptosis (Mortensen et al., 2023).

#### 2.2.2 Suppressors of lipid peroxidation

##### 2.2.2.1 The system X_c_^−^-GSH-GPX4 axis

The antiporter system X_c_^−^ is composed of two subunits, SLC7A11 and SLC3A2, and functions to import cystine into cells in exchange for glutamate. The cystine is degraded to cysteine, which is used to synthesize GSH (Liu et al., 2021). GPX4 is a selenoprotein that utilizes GSH to reduce lipid hydroperoxides, preventing lipid peroxidation and decreasing oxidative damage to the cells. There exist three isoforms of GPX4 localized to cytosol (cGPX4), nucleus (nGPX4) and mitochondria (mGPX4), respectively. GPX4 is unique among 8 known glutathione peroxides as it is the only enzyme capable of reducing oxidized fatty acids and cholesterol hydroperoxides. Mutations in GPX4 gene in humans led to cardiovascular, cerebrovascular, neuromuscular, or renal complications (Cheff et al., 2021). Deletion of GPX4, but not other GPX isoforms, caused embryonic lethality in mice (Yant et al., 2003; Yoo et al., 2012). Inducible ablation of GPX4 led to acute renal failure and early lethality in mice (Friedmann Angeli et al., 2014). Conditional deletion of GPX4 in neurons resulted in rapid onset of paralysis in the adult mice (Chen et al., 2015). GPX4 overexpression ameliorated, whereas GPX4 heterodeletion exaggerated myocardial ischemia/reperfusion (I/R) injury and doxorubicin-induced cardiomyopathy in mice (Miyamoto et al., 2022; Tadokoro et al., 2023).

##### 2.2.2.2 The GPX4-independent ferroptosis surveillance

**FIGURE 2:**
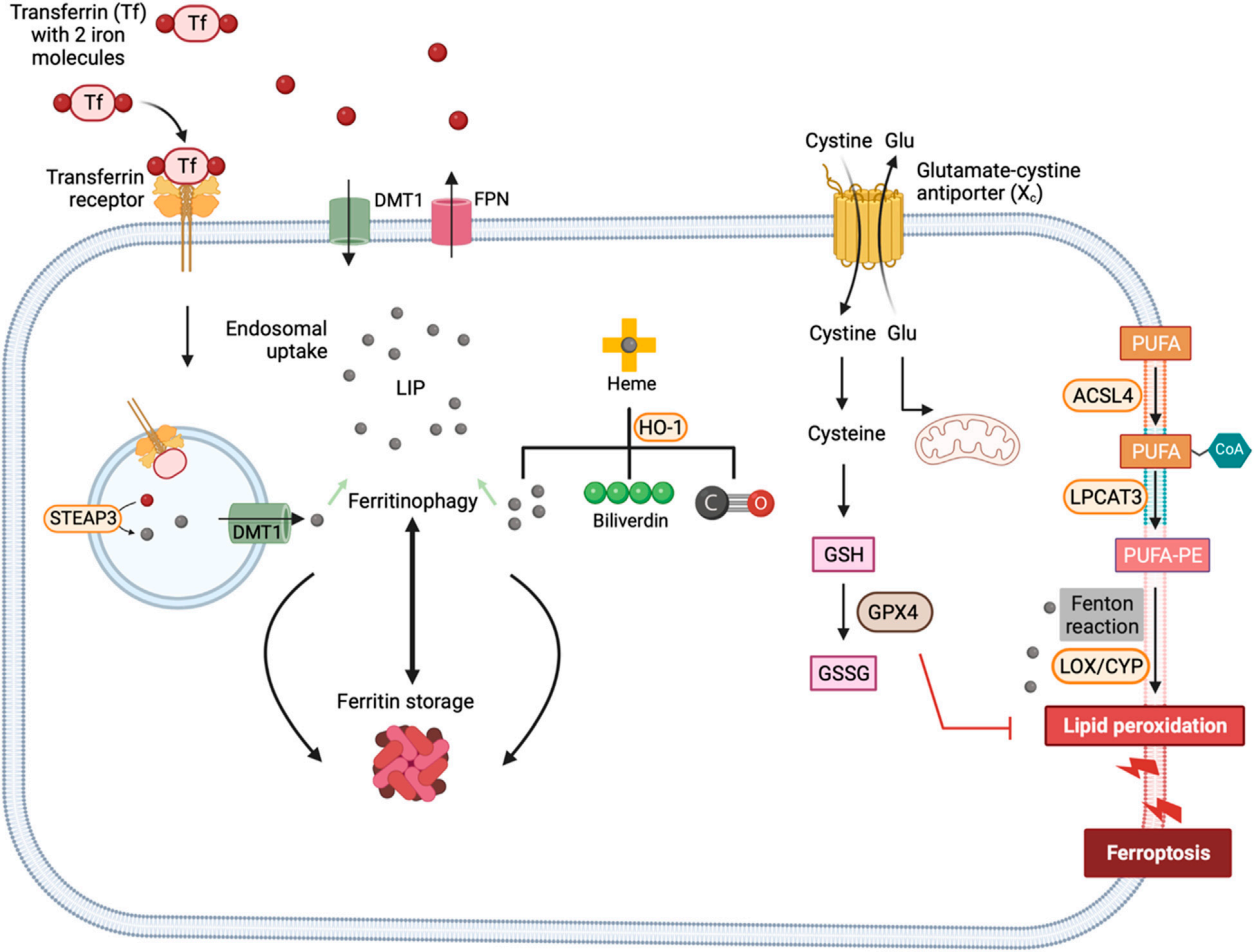
Mechanisms of ferroptosis. Ferroptotic cell death is triggered by iron-fueled excessive lipid peroxidation. Transferrin-TFR1 complex undergoes endocytosis and ferric iron is reduced to ferrous iron by the ferrireductase STEAP3. Iron is sequestered by ferritin or contribute to the LIP. HO-1 dependent heme degradation and NCOA4-mediated ferritinophagy also add iron to the LIP. Lipid peroxidation of PUFAs is mediated by the action of iron-dependent enzymes such as LOXs and CYP450 or iron catalyzed Fenton reactions. Glutamate cysteine exchanger mediates the exchange of extracellular cystine for intracellular glutamate. Once inside the cell, cystine is reduced to cysteine – a precursor for the synthesis of GSH. The activity of GPX4 depends on GSH to reduce lipid hydroperoxides and protect cell membranes from oxidative damage. ACSL4, Acyl-CoA synthetase long-chain family member 4; LPCAT3, Lysophosphatidylcholine acyltransferase 3; STEAP3, Six-Transmembrane Epithelial Antigen of the Prostate 3; DMT1, divalent metal transporter 1; LIP, labile iron pool; HO-1, hemeoxygenase-1; LOX, Lipoxygenase; CYP450, Cytochrome P450; GSH, Glutathione (reduced form); GSSG, Glutathione disulfide (oxidized form); GPX4, Glutathione peroxidase 4.

Ferroptosis suppressor protein 1 (FSP1, also known as AIFM2) has been identified as another key suppressor of ferroptosis. It converts ubiquinone (Coenzyme Q10) to ubiquinol (Coenzyme QH2), which effectively sequesters lipid peroxyl radicals (Doll et al., 2019). FSP1 is primarily a cytosolic protein and gets translocated to the plasma membrane following myristoylation of its N-terminal. Apart from its role in modifying ubiquinone, FSP1 also reduces vitamin K to its hydroquinone form, which acts as a potent antioxidant against lipid peroxidation (Mishima et al., 2022). Additionally, FSP1 contributes to the reduction of α-tocopheryl radicals to α-tocopherol, which serves as an effective scavenger of the lipid radicals. Interestingly, it has been shown that FSP1 mediates resistance against ferroptosis by recruiting endosomal sorting complexes required for transport (ESCRT)-III for repairing the cell membrane (Zeng et al., 2022). The cells are also equipped with other antioxidants such as vitamin E, thioredoxin and peroxiredoxins (Llabani et al., 2019; Kuang et al., 2020; Hu et al., 2021). Interestingly, nitric oxide (NO·) generated by inducible nitric oxide synthase (iNOS) has been shown to substitute GPX4 inactivity and suppress ferroptosis in macrophages (Kapralov et al., 2020). The major pathways that regulate ferroptosis are illustrated in Figure 2.

### 2.3 Role of key organelles in ferroptosis

#### 2.3.1 Mitochondria

Ferroptotic cells exhibit various aberrant morphological and functional changes in mitochondria including, decrease in cristae, reduced membrane potential, increased permeability, increased iron, ROS and lipid peroxidation, and elevated DNA stress (Dixon et al., 2012; Gan, 2021). Mitochondria depletion prevented ferroptosis induced by cysteine-deprivation or erastin (Gao et al., 2019). Moreover, mitochondrial DNA depletion or mitochondrial ROS quenching inhibited ferroptosis induced by RSL3 (Jelinek et al., 2018; Oh et al., 2022). Multiple mechanisms may contribute to mitochondria iron overload in ferroptosis. For example, increased mitochondrial iron uptake through iron transporters, such as mitoferrin-1 (SLC25A37) and mitoferrin-2 (SLC25A28), can mediate mitochondrial iron overload (Paradkar et al., 2009; Hung et al., 2013). Moreover, cytosolic iron is translocated into mitochondria via the mitochondrial Ca^2+^ and Fe^2+^ uniporter (MCU) in photodynamic therapy-induced ferroptosis, leading to mitochondrial iron overload (Shui et al., 2021). Defective heme biosynthesis in mitochondria can lead to iron accumulation. Heme synthesis is a multistep process that involves a sequential action of at least eight enzymes in mammals. It begins in mitochondrial matrix where 5- aminolevulinic acid (ALA) is produced by the action of aminolevulinic acid synthase (ALAS). Disruption of ALAS-dependent heme synthesis can impair iron utilization and trigger ferroptosis (Paradkar et al., 2009). Moreover, under the influence of different stressors, HO-1 can be upregulated and even translocated to mitochondria (Bindu et al., 2011; Bansal et al., 2014). Indeed, we recently found that oxidative stress promoted HO-1 translocation and mitochondrial iron overload (Chen et al., 2023b). Within mitochondria, Fe^2+^ is utilized for heme and Fe-S cluster synthesis or stored in mitochondrial ferritin (MTFT). Several proteins involved in mitochondrial iron metabolism have been implicated in defense against ferroptosis. Iron-sulfur cluster assembly scaffold protein (ISCU), for instance, plays a critical role in Fe-S cluster synthesis and overexpression of ISCU suppresses ferroptosis (Du et al., 2019). A cysteine desulfurase NSF1, which catalyzes the abstraction of sulfur from amino acid l-cysteine also protects against ferroptosis by preventing in mitochondrial iron overload (Alvarez et al., 2017). Another important protein, frataxin (FXN), is responsible for transferring iron to ISCU for the assembly of Fe-S clusters. Decreased FXN levels, as seen in Friedreich’s ataxia, result in mitochondrial dysfunction, iron accumulation, and ferroptosis (Cotticelli et al., 2019). Additionally, ABCB7 and ABCB8, members of ATP binding cassette (ABC) transporter family, are involved in exporting Fe-S clusters from the mitochondria to the cytosol, although their role in ferroptosis has not been directly examined (Guo et al., 2022). MitoNEET (also known as CISD1), a redox sensitive Fe-S cluster protein, regulates mitochondrial iron metabolism and ROS balance by interacting with transferrin receptor and voltage-dependent anion channel (VDAC) (Furihata et al., 2018; Lipper et al., 2019). Loss of CISD1 facilitates erastin-induced ferroptosis by increasing iron accumulation and oxidative stress in cancer cells (Yuan et al., 2016).

**FIGURE 3:**
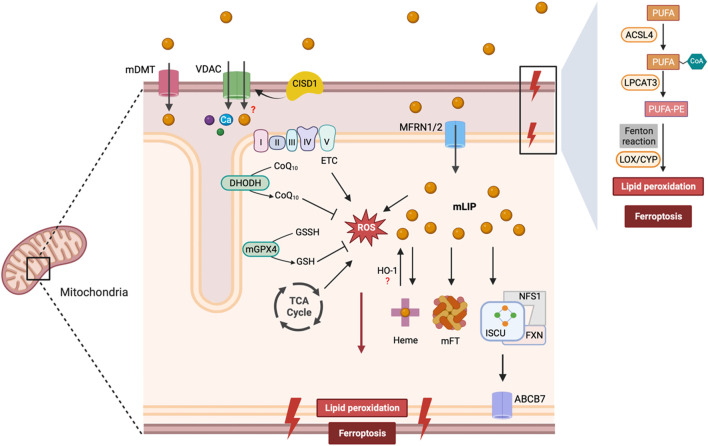
Mitochondrial regulation of ferroptosis. Iron is transported via mDT, MFRN1/2, and possibly VDAC into the mitochondria, where it is utilized for heme and Fe-S cluster synthesis. FXN transfers iron to ISCU and NFS1 serves as the sulfur donor for Fe-S cluster synthesis. Fe-S clusters are exported from the mitochondria to the cytosol via ABCB7 to allow the assembly of the cytosolic Fe-S containing proteins. Iron is sequestered by mitoferritin (mFT) or contributes to the labile iron pool within mitochondria (mLIP). Excess iron can lead to lipid peroxidation and ROS formation. ETC and TCA cycle also contribute to the mitochondrial ROS pool. GPX4 and DHODH represent two major antioxidant enzymes in mitochondria to prevent ferroptosis. Abbreviations: mDT, mitochondrial divalent transporter; VDAC, Voltage-Dependent Anion Channel; CISD1, CDGSH Iron Sulfur Domain 1; CoQ10, Coenzyme Q10; CoQ10H2, Coenzyme Q10 (reduced form); DHODH, Dihydroorotate Dehydrogenase; mGPX4, Mitochondrial Glutathione Peroxidase 4; GSSH, Oxidized Glutathione; GSH, Reduced Glutathione; MFRN1/2, Mitoferrin 1/2; mFT, Mitochondrial Ferritin; ISCU, Iron-Sulfur Cluster Scaffold Protein; FXN, Frataxin; ABCB7, ATP Binding Cassette Subfamily B Member 7; PUFA, Polyunsaturated Fatty Acids; ACSL4, Acyl-CoA Synthetase Long Chain Family Member 4; LPCAT3, Lysophosphatidylcholine Acyltransferase 3; LOX, Lipoxygenase; CYP, Cytochrome P450.

Mitochondria generate a significant amount of ROS at multiple sites such as, electron transport chain and tricarboxylic acid cycle, which interact with Fe-S clusters to release free iron and promote ROS generation via the Fenton reaction. Therefore, the combination of high iron levels and potential for ROS generation make mitochondria an optimal site for ferroptosis. Accumulation of mitochondrial lipid ROS has been detected in cells undergoing ferroptosis, while mitochondria-targeted ROS scavengers can inhibit ferroptosis in various cell types (Yamada et al., 2020; Jiang et al., 2022). On the other hand, mitochondria are also equipped with numerous antioxidant systems to combat ferroptosis (Ali et al., 2022). Several mitochondria-associated antioxidant proteins, including GPX4, SOD2, and MGST1, play a crucial role in protecting mitochondria from oxidative damage during ferroptosis (Chen J. et al., 2022). The inner membrane of mitochondria also functions as a site for synthesizing Coenzyme Q (CoQ) - a redox-active cofactor essential for FSP1 activity that provides protection against ferroptosis. Mitochondrial dysfunction has been associated with reduced levels of CoQ, which increases ferroptosis susceptibility (Mourier et al., 2015; Kühl et al., 2017). Mitochondrial dihydroorotate dehydrogenase (DHODH) has been shown to suppress ferroptosis by oxidizing DHO to orotate by using CoQ as electron acceptor (Mao et al., 2021). The role of mitochondria in ferroptosis is illustrated in Figure 3.

Accumulating evidence suggests that mitochondria-mediated ferroptosis contributes to the pathogenesis of heart disease. For example, mitochondria-mediated ferroptosis plays a key role in DOX-induced cardiomyopathy (Tadokoro et al., 2023). DOX downregulated GPX4 and induced excessive lipid peroxidation through DOX-Fe^2+^ complex in mitochondria, leading to mitochondria-dependent ferroptosis. Inhibiting ferroptosis by targeting mitochondrial-mediated pathways markedly attenuated DOX-induced cardiac toxicity (Fang et al., 2019). Mitochondria-mediated ferroptosis also mediates diabetic cardiomyopathy as well as catecholamine overload induced cardiomyopathy (Chen et al., 2023c; Chen et al., 2023a). Moreover, targeting mitochondrial ROS production effectively inhibits ferroptosis in cardiomyocytes (Sumneang et al., 2020; Chen et al., 2023a), offering a promising therapeutic option for the treatment of heart diseases by inhibiting ferroptosis.

#### 2.3.2 Lysosomes

Lysosomes play a crucial role in ferroptotic cell death through various mechanisms such as the activation of autophagy, release of lysosomal cathepsins, and accumulation of lysosomal iron or nitric oxide (Chen et al., 2021). Ablation of several autophagy related (ATG) genes, such as ATG3, ATG5, ATG6, ATG7, ATG8 and ATG13, has been shown to suppress ferroptosis, while activation of selective autophagy pathways prompts ferroptosis by targeting different cargoes. These include nuclear receptor coactivator 4 (NCOA4)-mediated ferritinophagy, sequestosome 1-mediated SLC40A1 degradation, chaperone-mediated autophagy (CMA) of GPX4, lipophagy-dependent breakdown of lipid droplets and mitophagy-mediated mitochondrial degradation (Wu et al., 2019; Liu et al., 2020; Li et al., 2021; Rizzollo et al., 2021; Bengson et al., 2023). Moreover, signal transducer and activator of transcription 3 (STAT3) has been shown to mediate erastin-induced ferroptosis through activation of cathepsin B. In contrast, pharmacological inhibition of lysosomal enzymes such as cathepsins and vacuolar H^+^ ATPase suppresses erastin-induced ferroptosis (Gao et al., 2018). These findings suggest that lysosomal pathways are important mediators and potential molecular targets of ferroptosis.

Lysosome-mediated ferroptosis has been shown to mediate the pathogenesis of heart failure. Lysosomal function is essential for intracellular iron metabolism. Lysosomal damage promotes the accumulation of iron and lipid peroxides, leading to the activation of ferroptosis. Improving lysosomal ferroptosis protected against heart failure in a mouse model with cardiomyocyte-specific knockout of the mitochondrial translation factor p32 (Yagi et al., 2023).

#### 2.3.3 Endoplasmic reticulum

The ferroptosis-inducing agents such as Erastin and RSL3 have been shown to trigger endoplasmic reticulum (ER) stress (Lee et al., 2018; Shin et al., 2018). ER stress plays a critical, yet complex role in regulating ferroptotic cell death via the eukaryotic translation initiation factor 2A (EIF2A)/activating transcription factor 4 (ATF4) pathway (Dixon et al., 2014). ATF4 inhibits ferroptosis by increasing the stability of GPX4 via HSPA5 upregulation or by promoting the expression of SLC7A11. Moreover, ER stress promotes membrane repair during ferroptosis via Ca^2+^-mediated ESCRT III activation. In contrast, ATF4 can upregulate ChaC glutathione-specific gamma-glutamylcyclotransferase 1 (CHAC1) expression which in turn degrades GSH, thereby contributing to ferroptosis (Chen et al., 2017; Wang N. et al., 2019). ER can suppress PUFA-mediated ferroptosis by promoting the biosynthesis of MUFA primarily catalyzed by the ER enzyme - stearoyl-CoA desaturase (SCD) (Sen et al., 2023). ER also regulates ferroptosis sensitivity, potentially via STING1-dependent autophagy or mitochondrial fusion (Smith, 2021; Zhang Z. et al., 2022).

It has been shown that Inhibition of endoplasmic reticulum stress could alleviate ferroptosis and cell injury (Li W. et al., 2020). CHOP-mediated ER stress has also been shown to play an important role in I/R injury (Dixon et al., 2014). Moreover, iron overload in the ER triggers ferroptosis during cardiac I/R injury (Miyamoto et al., 2022). Therefore, these studies suggest that ERS induced by ferroptosis contributes to the pathogenesis of cardiac I/R injury.

### 2.4 Crosstalk between ferroptosis and other cell death pathways

Emerging evidence reveals that ferroptosis may crosstalk with other cell death pathways. Ferroptosis is a type of autophagy-dependent cell death (Zhou et al., 2020). Ferroptosis inducers promote the activation of autophagy, leading to the accumulation of autophagic vesicles. NCOA4-mediated autophagy, termed ferritinophagy, induces ferritin degradation and iron overload, promoting oxidative stress and ferroptosis. Elevated lipid peroxidation in ferroptosis also promotes GSDMD-mediated pyroptosis. Indeed, it has been shown that deletion of GPX4 led to lipid peroxidation-dependent caspase 11 and GSDMD cleavage (Kang et al., 2018). Elevated mitochondrial ROS during ferroptosis may also promote necroptosis, possibly by increasing the autophosphorylation of RIPK1 (Zhang et al., 2017). Moreover, the release of damage-associated molecular pattern molecules (DAMPs) from the plasma membrane pore is a common feature of necrotic cell death such as ferroptosis, pyroptosis, and necroptosis. The release of the DAMPs triggered by ferroptosis may further promote pyroptosis and necroptosis. The significance of ferroptosis-pyroptosis crosstalk in heart disease needs to be further investigated.

## 3 Ferroptosis in heart diseases

### 3.1 Myocardial ischemia/reperfusion injury

Myocardial ischemia/reperfusion (I/R) injury can occur during the restoration of blood supply to the acutely ischemic heart and contributes to the final infarct size (Zhang et al., 2023). Ferroptosis has recently been implicated in the pathogenesis of myocardial I/R injury (Han et al., 2023). I/R injury causes iron overload characterized by increased cardiac nonheme iron levels and ferritin expression (Fang et al., 2019). During I/R injury, there is also a time-dependent increase in ACSL4 levels with a concomitant decrease in GPX4 activity (Tang L.-J. et al., 2021). Pharmacological inhibition of ferroptosis with ferrostatin-1 or dexrazoxane has been shown to reduce cardiac infarct size following I/R (Fang et al., 2019). Moreover, inhibiting ferroptosis can also provide long-term benefits against I/R-induced cardiac remodeling and fibrosis. In patients undergoing coronary artery bypass grafting (CABG) surgery, infusion of an iron chelator deferoxamine also suppressed reperfusion-induced oxidative damage (Paraskevaidis et al., 2005).

### 3.2 Heart failure with preserved ejection fraction (HFpEF)

Iron overload has been linked to endothelial dysfunction, impaired excitation-contraction coupling of cardiomyocytes, myocardial inflammation and tissue fibrosis, which all contribute to the development of HFpEF (Li et al., 2022). A recent study reveals that obesity-induced HFpEF leads to an upregulation in iNOS activity while reducing GPX4 activity. Further, treatment with an anti-diabetic agent, Imeglimin has been shown to prevent HFpEF by inhibiting myocardial production of iNOS and restoring myocardial expression of GPX4 (Kitakata et al., 2021). Tandem Mass Tag (TMT)-based proteomics studies reveal that ferroptotic metabolic pathways contribute to the development of HFpEF (Ma et al., 2022). Additionally, rats with HFpEF exhibited an increase in Fe^2+^ concentration and lipid peroxidation products, accompanied by increased expression of TFR1 and ACSL4 proteins. Moreover, there was a significant decrease in GSH concentrations and downregulation of xCT and FTH1 expression in HFpEF (Ma et al., 2022). These findings suggest that ferroptosis may contribute to the pathogenesis of HFpEF.

### 3.3 Hypertrophic cardiomyopathy (HCM)

Dysregulation of iron metabolism and increased lipid peroxidation have been implicated in cardiac hypertrophic remodeling (Tang et al., 2019; Fan et al., 2022). Recent studies further revealed that ferroptosis plays a role in hypertrophic cardiomyopathy. It has been shown that xCT, a key regulator of ferroptosis, prevents cardiac hypertrophy by inhibiting ferroptosis (Zhang X. et al., 2022). xCT knockout aggravated angiotensin II (Ang II)-induced cardiac hypertrophy, fibrosis, and dysfunction (Zhang X. et al., 2022). Similarly, loss of ferritin H, a key iron storage protein, led to hypertrophic cardiomyopathy by inducing cardiac ferroptosis, which was rescued by overexpression of xCT (Fang et al., 2020). Moreover, overexpression of TRIM44, a deubiquitinase, promoted pressure overload-induced cardiac hypertrophy via activation of TLR4/NOX4-mediated ferroptosis (Wu et al., 2023).

### 3.4 Doxorubicin-induced cardiomyopathy

Doxorubicin (DOX) induces cardiotoxicity, referred to as DOX-induced cardiomyopathy, which limits its clinical use as a chemotherapeutic agent (Rawat et al., 2021). The mechanism of DOX-induced cardiomyopathy remains incompletely understood, but recent studies have highlighted a prominent role of ferroptosis in pathogenesis. Fang et al. identified ferroptosis as a key mechanism for DOX-induced cardiomyopathy in mice (Fang et al., 2019). Importantly, inhibition of ferroptosis with ferrostatin-1 and dexrazoxane effectively attenuated DOX-induced cardiomyopathy. Mechanistically, they revealed that mitochondrial iron overload and lipid peroxidation play a key role in DOX-induced myocardial ferroptosis. Tadokoro et al. also showed that mitochondria-dependent ferroptosis plays a key role in DOX cardiomyopathy (Tadokoro et al., 2023). GPX4 expression was markedly downregulated during DOX cardiomyopathy, accompanied by increased lipid peroxidation in mitochondria. Importantly, transgenic overexpression of GPX4 ameliorated, whereas heterodeletion of GPX4 exacerbated DOX cardiomyopathy. Abe et al. further showed that DOX induces mitochondria-dependent ferroptosis by intercalating into mitochondrial DNA (mtDNA) (Abe et al., 2022). Moreover, DOX also disrupts heme synthesis and impairs iron utilization by downregulating 5′-aminolevulinate synthase 1 (Alas1), leading to mitochondrial iron overload and ferroptosis.

### 3.5 Other cardiomyopathies

Ferritinophagy-mediated ferroptosis has been shown to contribute to the pathogenesis of septic cardiomyopathy (Li N. et al., 2020). Recent findings reveal a role of islet cell autoantigen 69 (ICA69)-STING signaling and transmembrane protein 43 (TMEM43) in lipopolysaccharide (LPS)-induced cardiomyocyte ferroptosis and cardiomyopathy. Ablation of ICA69 decreased STING trafficking and improved overall cardiac function by targeting LPS-induced ferroptosis. ICA69 levels are also positively correlated with the severity of sepsis in humans (Kong et al., 2022). Overexpression of TMEM43 inhibited LPS-induced ferroptosis with increased levels of SLC7A11 and GPX4, revealing a protective role of TMEM43 against sepsis-induced cardiomyopathy (Chen Z. et al., 2022).

A growing body of evidence highlights the role of ferroptosis in diabetic cardiomyopathy (DCM). The advanced glycation end-products (AGEs) that accumulate in cardiac tissue with the onset of diabetes, can induce ferroptosis as evident by increased MDA levels, upregulation of COX2, and downregulation of ferritin and SLC7A11. Moreover, activation of AMPK/NRF2 pathways with sulforaphane protects heart against AGE-induced ferroptosis (Wang X. et al., 2022). In contrast, Nrf2 signaling can also exert detrimental effect to the heart, particularly when autophagy is impaired such as in chronic in type 1 diabetes (Zang et al., 2020).

Ferroptosis has recently emerged as a potential contributor to radiation-induced cardiomyopathy (RICM) (Wang B. et al., 2020). Radiation exposure induces ROS production, which triggers lipid peroxidation and subsequent ferroptosis (Lei et al., 2020). Endothelial cell injury caused by radiation is an early event in RICM, leading to the release of cytokines and chemokines such as IL-6, IL-8, TGF-β, TNF-α, and IL-1β (Li X. et al., 2019; Li W et al., 2019; Wang C. et al., 2020). Increased ROS production and lipid peroxidation further contribute to endothelial cell damage, myocardial fibrosis, and cardiomyopathy (D’Oria et al., 2020; Jiang et al., 2021). Activation of the STING pathway and subsequent induction of interferon gamma and COX2 expression have been observed following radiation exposure (Lemos et al., 2020; Storozynsky and Hitt, 2020). Moreover, damaged endothelial cells release danger-associated molecular patterns (DAMPs), such as high mobility group box 1 (HMGB1), which promote ferroptosis and inflammation (Dyer et al., 2018; Zhou et al., 2018; Green, 2019).

### 3.6 Targeting ferroptosis in heart disease

Ferroptosis as a potential target for the treatment of heart disease has been explored in various experimental models. Genetic or pharmacologic inhibition of ferroptosis has been shown to illicit cardioprotective effects in these studies (Table 1). Therefore, anti-ferroptosis therapies may hold a tremendous promise for the treatment of heart disease in humans. Several drugs currently in clinical use have been shown to target ferroptosis. For example, dexrazoxane (DXZ) has been used to treat doxorubicin-induced cardiotoxicity, which reverses DOX-induced ferroptosis mainly by chelating mitochondrial iron (Ichikawa et al., 2014). Several other iron chelators, including deferoxamine (DFO), deferiprone (DFP), and deferasirox (DFX) are clinically approved for managing iron overload-related diseases. In addition, N-acetylcysteine (NAC) has been shown to inhibit ferroptosis by targeting cysteine metabolism. NAC has been clinically shown to improve neurodegeneration-related symptoms by increasing cysteine levels and facilitating the synthesis of GSH (Monti et al., 2016). Notably, edaravone, a radical-trapping antioxidant clinically approved for treating acute ischemic stroke and amyotrophic lateral sclerosis, has been shown to inhibit ferroptosis under various pathological conditions (Homma et al., 2019). Thiazolidinediones (TZDs), such as rosiglitazone, pioglitazone, and troglitazone, are approved to treat adult type 2 diabetes, which have suppressing ferroptosis activity by selectively inhibiting ACSL4 (Doll et al., 2017). Of note, a screening of a library consisting of FDA-approved drugs has led to the successful identification of multiple ferroptosis inhibitors (Tan et al., 2024), which offers new therapeutic possibilities for the treatments of ferroptosis-related diseases.

**TABLE 1:** Targeting ferroptosis in experimental models of heart disease.

Pharmacologic/genetic manipulation	Disease model	Phenotypes	References
Deferoxamine	I/R injury (*in vivo*)	↓ IR injury	Tang et al. (2021b)
Dexrazoxane Ferrostatin-1		↓ infarct size ↓ cardiac remodeling	Fang et al. (2019)
Zileuton		↓ infarct size ↓ tissue injury	Gonca (2017)
ABCB8 TG		↑ cardiac function ↓ cardiac remodeling	Chang et al. (2016)
2,2′-bipyridyl		↑ cardiac function ↓ cardiac remodeling	
Dexmedetomidine	I/R injury (*ex vivo*)	↓ infarct size	Wang et al. (2022b)
Liproxstatin-1		↓ infarct size	Feng et al. (2019)
mGPX4 TG		↑ cardiac function ↓ cardiac injury	Dabkowski et al. (2008)
FTH1 KO	Hypertrophic cardiomyopathy	↑ cardiac remodeling	Fang et al. (2020)
xCT KO		↑ cardiac remodeling	Zhang et al. (2022a)
NCOA4 KO		↓ cardiac remodeling	Ito et al. (2021)
Dexrazoxane Ferrostatin-1 Mito TEMPO Zinc protoporphyrin IX	DOX-induced cardiomyopathy	↑ cardiac function ↓ cardiac remodeling ↓ cardiac injury	Rocha et al. (2016), Fang et al. (2019)
GPX4 TG		↑ cardiac function ↓ myocardial atrophy	Tadokoro et al. (2020)
GPX4 KO		↑ cardiac function ↑ myocardial atrophy	Tadokoro et al. (2020)
FUNDC2 KO		↑ cardiac function ↓ cardiac remodeling	Ta et al. (2022)
Ferrostatin-1	Post-transplant cardiomyopathy	↓ cardiac remodeling	Li et al. (2019a)
MitoTEMPO	Diabetic cardiomyopathy	↓ tissue remodeling ↑ cardiac function	Ni et al. (2016)
Pioglitazone		↑ cardiac function ↓ cardiac injury	Clarke et al. (2017), Doll et al. (2017)
Deferiprone Dexrazoxane Ferrostatin-1	Sepsis-induced cardiac injury	↓ tissue injury ↑ cardiac function ↑ cell survival	Li et al. (2020a)

The clinical application of ferroptosis-related targets is still in its infancy. Iron metabolism-related indicators, such as serum iron, serum ferritin, transferrin and soluble transferrin receptors, have been used to monitor the progression of heart disease. For example, patients with elevated serum ferritin showed a higher incidence of acute myocardial infarction than those with reduced serum ferritin (Moradi et al., 2015). Moreover, elevated levels of soluble ferritin receptors corelates with a higher risk of coronary atherosclerotic heart disease (Braun et al., 2004). Hepcidin concentration has also been used to predict the risk of myocardial infarction or cardiovascular death (Zeller et al., 2018). Notably, elevated levels of ferritin and hepcidin were associated with a higher risk of heart failure in women (Klip et al., 2017). Other biomarkers of ferroptosis, such as lipid peroxidation products, might also be useful in monitoring the progression of heart disease.

## 4 Conclusions and perspectives

Recent studies clearly demonstrate that ferroptosis contributes significantly to the pathogenesis of multiple forms of heart disease including acute cardiac injury and chronic disorders. Genetic or pharmacologic inhibition of ferroptosis showed beneficial effects under pathological conditions such as myocardial infarction and heart failure. Recent studies also provide new mechanistic insights into the regulatory mechanisms of ferroptosis, including new pathways that positively or negatively regulates ferroptosis signaling and the crosstalk between different subcellular compartments in orchestrating ferroptotic cell death. Numerous studies have demonstrated that cardiomyocytes undergo ferroptosis in response to pathological stress *in vivo* and *in vivo*. Ferroptosis of other cell types in the heart, including endothelial cells, smooth muscle cells and macrophages, has also been shown to play a role in the pathogenesis of certain forms of heart disease (Leng et al., 2021). The relative contribution of ferroptosis in different cell types to disease pathogenesis warrant further investigation under various disease conditions. Notably, interaction between ferroptosis and other modes of cell death, such as pyroptosis, necroptosis, and autophagy, increases the complexity of these pathways. Various cell death processes contribute to the loss of cardiac cells in heart diseases, and their specific roles in disease pathogensis need further investigation. It will be important to develop new diagnostic tools for assessing ferroptosis *in vivo*, given the lack of reliable and specific biomarkers for ferroptosis. Targeting ferroptosis represents an important therapeutic opportunity for the treatment of heart disease. Pharmacological inhibitors of the ferroptosis pathway have been developed for use in experimental settings, such as iron chelators and lipophilic antioxidants. However, given that adverse effects have been observed with these compounds (Miller et al., 2005; Tebbi et al., 2007), the identification of new molecular targets of the ferroptosis pathway and the development of novel ferroptosis inhibitors will be important for anti-ferroptosis therapies. Targeting the ferroptosis pathway represents a promising therapeutic strategy for various forms of heart disease, although these approaches warrant further investigation in clinical studies.
